# Suicidality Prevalence in a Pediatric Psychiatric Clinic: Relation to Social and Environmental Risk Factors

**DOI:** 10.3390/children10030558

**Published:** 2023-03-15

**Authors:** Stavroula Ilia, Evangelia Sakapeti, Panagiotis Briassoulis, George Gerostergios, Alexandros Vgontzas, George Briassoulis

**Affiliations:** 1 Postgraduate Program “Emergency and Intensive Care in Children Adolescents and Young Adults”, School of Medicine, University of Crete, 71003 Heraklion, Greece; 2 Pediatric Intensive Care Unit, University Hospital, School of Medicine, University of Crete, 71110 Heraklion, Greece; 3 Department of Psychiatry, University Hospital, School of Medicine, University of Crete, 71110 Heraklion, Greece; 4 Attikon University Hospital, School of Medicine, National and Kapodistrian University of Athens, 12462 Athens, Greece

**Keywords:** children, pediatrics, pediatric intensive care, critical care, psychiatric, suicidality, stressful, social, stress

## Abstract

Suicidality is a growing public health problem in children and adolescents. The aim of this retrospective data analysis study was to estimate the prevalence of suicidality in pediatric patients admitted to an academic Pediatric Psychiatric Clinic (PPC) and to analyze social and environmental risk factors associated with suicide. Suicidal ideation was assessed by the Self-Injurious Thoughts and Behaviors Interview. Using established psychometric scales, social and stressful events were analyzed. During the four-year study, 249 episodes of care were experienced by 152 individuals (mean age 15.2 ± 2 years, girls/boys 107/45). Twenty-eight patients (11.2%) were admitted from the Pediatric Intensive Care Unit and the Department of Pediatrics, 162 (65.1%) from the Pediatric Emergency Department, and 59 (23.7%) from other Hospitals (*p* = 0.003). A significant longitudinal increase in admissions to PPC, with increasing trends of suicidal ideation, suicide attempts, and suicidality, was recorded. Suicidal behavior, bullying, internet addiction, friends quarreling, and family problems were risk factors for suicide attempts and suicidality. Our results have implications for prevention programs, highlighting an increasing need for care for suicide attempts and suicidal ideation, related to specific stressful events and contextual socio-environmental status.

## 1. Introduction

Suicidality is defined by the American Psychological Association as “the risk of suicide, usually indicated by suicidal ideation or intent, especially as evident in the presence of a well-elaborated suicidal plan, including suicidal thoughts, plans, gestures, or attempts [1]”. Suicidal ideation refers to thoughts of death. The severity of suicidal ideation can vary from fleeting and unwanted thoughts to a preoccupation with death that may involve detailed planning [2].

Suicide is a leading cause of death in youth worldwide and the second cause among children and adolescents 10–24 years old. In a 5-year retrospective study of psychiatric hospitalizations of adolescents, half of the participants had experienced stressful events during childhood, with the most frequent admission reasons being aggressive behavior in males and suicide risk in females [3]. Associations between traumatic events and suicidal behavior could be used to develop more personalized intervention strategies aimed at improving mental and behavioral health of adolescents [4]. In addition, depression and anxiety in children positively predicted their suicide ideation [5].

An increase in admissions has been also recorded, affecting adolescents with suicidality/self-harm and substance-related disorders [6]. Importantly, 78.2% of adolescent admissions were due to suicidal behavior associated with family risk factors and life stressors [7]. The coronavirus disease 2019 (COVID-19) pandemic is a recent example of a mental threat to vulnerable populations, especially children and adolescents [8]. Increased psychological stress during the pandemic was associated with an increased risk of active suicidal ideation and its severity [9]. Longitudinally, the pandemic influenced the type and complexity of mental health problems, increasing the diversity and severity of suicidal behaviors and the frequency of counseling for psychotic symptoms [10]. The presence of passive suicidal ideation has been recently reported in 10.5% of 9-year-old children [11]. A recent study in children 9–10 years old demonstrated that high family conflict and low parental monitoring were associated with suicidal ideation, suicide attempts, and non-suicidal self-injury [12].

Over the past decade, visits to American and Canadian psychiatric emergency departments for child and youth life-threatening concerns have increased substantially [13]. In a 5-year period in a Pediatric Psychiatric Clinic (PPC) in Italy, a higher admission rate for females was recorded, with an average age of 13.4 years. Most of the admissions occurred through the Pediatric Emergency Department (PED), and suicidal behavior was the most frequent reason for admissions [14]. During a prolonged economic crisis, overall suicide mortality rates in Greece have increased by 40%, and Crete has been highlighted as the island with the most worrying increase, with females showing the highest rise [15,16].

Under current circumstances, investigating PPC admissions might be a noble and interesting direction for challenging research. We hypothesized that it would be of great importance to investigate the prevalence of active suicidal ideation, suicide attempts, and their associated risk factors in such a high-risk population with the aim of preventing an expanding life-threatening public health concern. Accordingly, the purpose of this study was: (1) to study suicide-related admissions in pediatric patients admitted to a PPC through the PED, Pediatric Intensive Care Unit (PICU), or other departments; (2) to analyze individual, familial, social, and environmental risk factors and their relations to suicidality in this cohort of patients; (3) to assess recent stressful events and longitudinal trends of suicide attempts, active suicidal ideation, and overall suicidality in a 4-year period.

## 2. Materials and Methods

### 2.1. Study Design

This retrospective data analysis research was conducted in the PPC, located in an academic Hospital, responsible for treating psychiatric emergencies. The sample was constituted of all children and adolescents (age 5–18-years-old) who accounted for acute hospitalizations in the PPC from February 2015 to September 2018. Exclusion criteria were patients younger than 5 or older than 18-years-old; those who did not have a parent who completed the Network of Relationships Inventory or relevant psychometric scales; and patients with parents who were not sufficiently fluent in Greek. Additionally, patients who presented with non-suicidal self-injury (NSSI) were admitted to the surgical departments and excluded from this study.

### 2.2. Setting

The mental health inpatient unit at the PPC in Heraklion, Crete, is an accredited facility that accommodates ten patients aged 5–18-years-old suffering from major mental, psychological, emotional disorders, and/or behavioral alterations that cannot be treated in an outpatient setting. Medical or life-threatening complications due to acute psychiatric conditions are initially managed in the PICU; patients are transferred to the PPC when stable. Upon discharge, the patient is sent back home with a consultation of the referring pediatric psychiatrist to maintain an uninterrupted treatment.

### 2.3. Ethical Approach

Anonymity was ensured and patients’ names were replaced by a unique identification number. This study was conducted in accordance with the principles of the Declaration of Helsinki (World Medical Association Declaration of Helsinki, 1964) and was not sponsored by any pharmaceutical company. This study received ethical approval from the Institutional Review Board of the University Hospital (ID 11927/12/9/2014). Formal patient and/or parental consent was waived by the review board—in view of the retrospective and anonymized analysis of the data.

### 2.4. Patients

For each patient enrolled in the study, de-identified demographic, social, and psychopathological data were recorded. Age, gender, educational and school performance (cumulative grade point average), history of a previous mental health disorder, social problems, and history of physical or sexual abuse were collected. Parental disorders, including psychiatric diseases or addictions, and parents’ relationship were also recorded. Data from reports of pediatric psychiatrists and psychologists were added. The following data were collected by reviewing medical records: admission reason; modality (voluntary or involuntary); outset of psychiatric pathology and recent life stressful events as previously described; the presence of suicidal ideation, suicide attempt, suicide attempt method, aggressive behavior, family, friends, and social environment. Longitudinal trends in suicidality rates were assessed by sex and age group (5–10, 11–14, and 15–18-years-old).

### 2.5. Suicidality

In this study, suicidality covered active suicidal ideation (serious thoughts about taking a patient’s own life, suicide plans) and suicide attempts. Suicidal ideation was assessed upon admission by the Self-Injurious Thoughts and Behaviors Interview [17].

### 2.6. Recorded Psychometric Scales

Recorded responses to established psychometric scales about social behavior, family structure, and lifetime use of illegal drugs [18] were recorded from the medical records. Any lifetime use of illegal drugs was established for each substance separately. Then, for the needs of this study, use of tobacco was defined as smoking at least six cigarettes per day in the last 30 days; use of alcohol was defined as drinking at least 10 times in the last 30 days; use of cannabis was defined as reporting any use in the last 30 days; any lifetime use of tranquillizers and sedatives without prescription was defined as the use of available commercial names of tranquillizers and sedatives; use of any other illegal drug was defined as any lifetime use of cocaine or heroin, ecstasy, crack, amphetamines, and hallucinogens [18]. Stressful events such as migration; bullying; abuse of any etiology; and family, friend, or school problems were analytically documented. The “antisocial behavior” consisted of a 10-item scale of frequencies relating to causing damage to property, being involved in fights, and theft in the last year. The socio-economic status (total household net income, parental education and occupation, and material wealth) was collected from the patients’ demographic records and recoded into three categories (high, middle, and low) [19].

A patient who had fulfilled any five of the following reported eight adapted criteria—using the internet as a means of regulating mood; putting a job or relationship in jeopardy to use the internet; lying to others about how much time is spent online; irritability, depression, or mood lability when internet use is limited; repeated efforts to curtail internet use; preoccupation with the internet; staying online longer than anticipated; and a need for increased time spent online to achieve the same amount of satisfaction—was regarded as internet-addicted [20]. Information regarding the parent–child relationship was taken from the answers to the Network of Relationships Inventory, which was used to assess quality using a 13-item short form [21]. The inventory scales of the form assess the extent to which attachment behaviors, caregiving behaviors, companionship, affiliative behaviors, conflicts, antagonism, and criticism occur in the parent–child relationships.

### 2.7. Associated Predictors

The associated predictors analyzed were family or school stressful events, quarreling with friends, bullying, internet addiction, physical or sexual abuse, substance abuse, and clinical or social environment and characteristics. Parental disorders, including psychiatric diseases or addictions, and parents’ relationships were also recorded. Parental psychiatric diagnoses were identified based on at least two outpatient or one inpatient claims records, based on the International Classification of Diseases (tenth revision (ICD-10)) codes [22]. Parents diagnosed with any psychiatric disorder, such as schizophrenia, bipolar spectrum disorders, depressive disorders, anxiety disorders, obsessive–compulsive disorder, attention deficit hyperactivity disorder, autism spectrum disorder, adjustment disorders, and substance use disorders, were defined as having a psychiatric disorder. Parents’ relationships were recorded from family social interviews, as biological parents who remained married, biological parents who divorced, step-parents, and unmarried biological parents. Data from reports of pediatric psychiatrists and psychologists were added.

### 2.8. Statistical Analysis

To calculate an adequate sample size, we used the G*Power statistical power calculator: chi-squared test fixed effects; power = 0.80, alpha = 0.05, effect size medium (f = 0.3). The calculated total sample size was 143. Repeated power analysis of ANOVA fixed effects, one-way; power = 0.80, alpha = 0.05, effect size medium (f = 0.3). The calculated total sample size for the cluster measurements was 195 records. The Shapiro–Wilk test was used to assess the normality of the distribution. Categorical variables are described in absolute values and frequency. Quantitative variables are expressed in mean and standard deviation. For each variable, the frequency of occurrence in the corresponding sample set was calculated. An ANOVA test was used to compare quantitative variables, and the chi-squared test, corrected by Fisher’s exact test, was used for statistically significant differences in the frequencies of all variables by admission year, age group, gender, and suicidality or suicidal attempt. A logistic regression model (backward stepwise (Likelihood Ratio) method) was adopted to examine whether any of the studied risk factors are independently associated with suicidality. To evaluate risk factors’ independent prediction ability, the areas under the receiver operating characteristic curves (AUROC) for risk factors significantly associated with suicide attempts were calculated. Data were analyzed by using the SPSS v.28 statistical package. Statistical significance was set at *p* < 0.05.

## 3. Results

### 3.1. Patients

**Figure 1:**
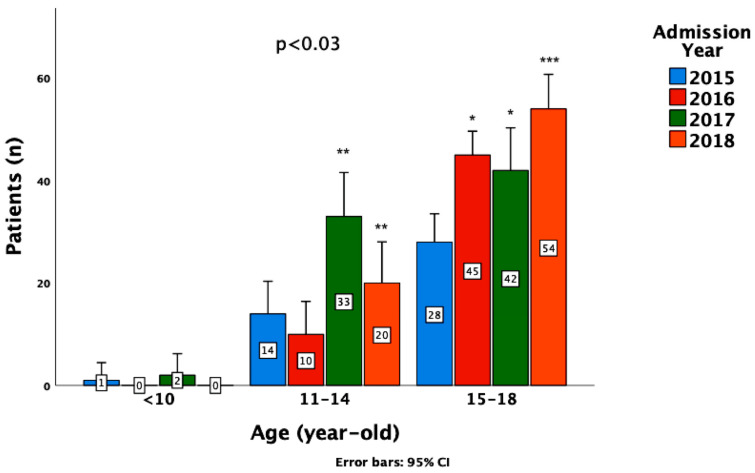
Longitudinal trends of admissions to the Pediatric Psychiatric Clinic per age group (ANOVA test, *p* < 0.03). Post hoc tests (Bonferroni, *p* < 0.05): Admission differences between * 2016 or 2017 vs. 2015; ** 2017 or 2018 vs. 2015 and 2016; *** 2018 vs. 2015, 2016, and 2017.

A total of 249 admissions of 152 individuals (mean age 15.2 ± 2 years, mean length of stay 24 ± 31 days) were recorded and studied. One-hundred and fifty-two (61%) were first admissions and ninety-seven (39%) were repeat hospitalizations. Twenty-eight patients (11.2%) were admitted from the Pediatric Intensive Care Unit and the Department of Pediatrics, one-hundred and sixty-two (65.1%) from the Pediatric Emergency Department, and fifty-nine (23.7%) from other Hospitals (*p* = 0.003). A longitudinal increase in admissions to the PPC was recorded from 2015 to 2018 (*p* < 0.03), especially in the 15–18-year-old group (Figure 1).

**Figure 2:**
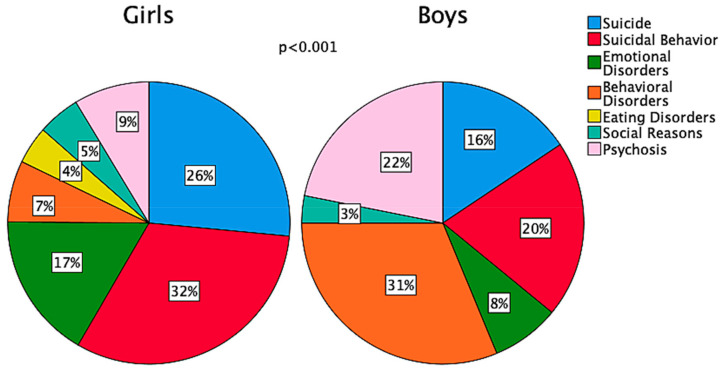
Sex-related distribution of reasons for admission to the Pediatric Psychiatric Clinic during the study period.

The reasons for admission to the PPC varied by sex (*p* < 0.001), with more than half of girls admitted for attempted suicide (26.5%) or suicidal behavior (32%), and most boys admitted for behavioral disorders (31%) and psychosis (22%) (Figure 2).

Among patients < 11-year-old, only behavioral or emotional disorders were recorded, whereas eating disorders were present only in the 11–14-year-old group (*p* < 0.002). The reasons for PPC referrals per age group, origin, admission diagnosis, and transportation conditions are shown in Table 1.

The most common discharge ICD-10 diagnoses were emotional disorders (36.5%) and behavioral and emotional disorders with onset in childhood and adolescence (17.2%), and were associated with active suicidal ideation and suicidality (*p* < 0.001). Other less frequent diagnoses were anxiety disorder and somatoform disorders in girls, and psychotic and developmental disorders in boys (*p* < 0.001). The main reasons for readmission to PPC were attempted suicide (27%) or suicidal behavior (22.4%).

### 3.2. Social and Family Characteristics

The basic characteristics of patients admitted to PPC during the study period stratified by age group are presented in Table 2.

More boys (36%) dropped out of abounded at school compared to girls (16%). Stressful events in the family (34.1% vs. 21.9%) mostly influenced girls (*p* < 0.046), whereas quarreling with friends did not differ between girls (24.9%) and boys (17.2%). The longitudinally-increased internet addiction (from 14.3% to 40.7%, *p* = 0.016) was most prevalent among males (32 (50%) vs. 59 (31.9%), *p* < 0.08) More girls were victims of physical (20 vs. 5) and sexual (14 vs. 1) abuse (*p* = 0.03) or bullying (26% vs. 19%) compared to boys (*p* = 0.16).

### 3.3. Suicidality

**Figure 3:**
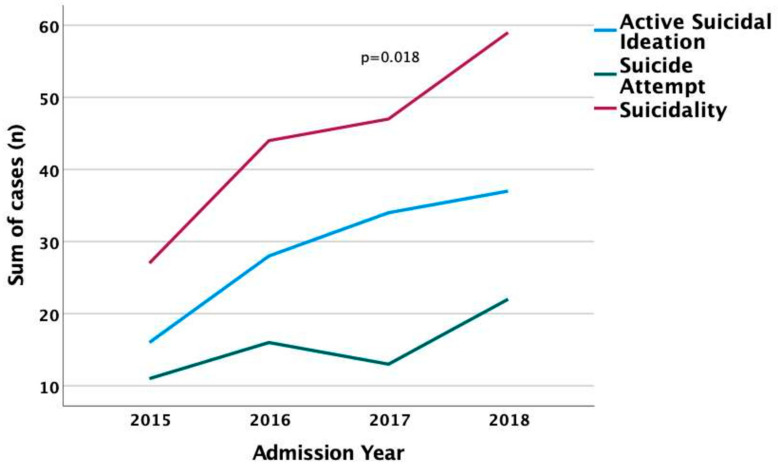
Suicidality, suicidal ideation, and suicide attempt annual rates during the 4-year study period.

A large proportion of patients hospitalized in the PPC expressed suicidality (177/249, 71.1%). In all admissions, suicidality was more prevalent in girls (137/185, 74.1%) compared to boys (40/64, 62.5%, *p* = 0.05), with a similar trend in suicidal ideation (47% vs. 43%, *p* = 0.38) and suicide attempts (27% vs. 18.8%, *p* = 0.124). Suicidality annual rates, expressing suicide attempt rates, and active suicidal ideation longitudinal trends increased significantly (*p* = 0.018) (Figure 3).

### 3.4. Risk Factors Associated with Suicidality

Suicide attempts in the past (41.9%, *p* < 0.001) and self-harm behavior (38.3%, *p* < 0.001) were strongly associated with suicidality. Recent stressful events associated with suicidality were problems with family relationships (35.6%, *p* = 0.015), quarreling with friends (29.9%, *p* < 0.001), internet addiction (41.2%, *p* = 0.02), and bullying (32.2%, *p* < 0.001). Chronic pathologic conditions, substance abuse, physical and sexual abuse, and parental negative relations or psychiatric disorders were not related to suicidality.

In a logistic regression model (backward stepwise (Likelihood Ratio) method), the following risk factors were independently associated with suicidality: friends quarreling (odds ratio for suicidality 11.3, *p* < 0.001), bullying (7.4, *p* = 0.001), family problems (3.3, *p* = 0.002), and internet addiction (2.76, *p* = 0.005).

### 3.5. Suicide Attempt

Neither suicidality nor suicidal ideation or suicide attempts differed among age groups (Table 3). Suicide attempts recorded during the first admission (*n* = 43) or readmission (*n* = 19), along with reported previous attempts of admitted patients, gave an aggregate of 109 lifetime suicide attempts among 152 patients (71.7%) in 249 admissions (43.8%). The ways of suicide attempts did not differ between sexes, age groups, or longitudinally (Table 3).

However, younger patients more often jumped from height or cut their wrists compared to older adolescents. Patients with recorded self-harm behavior with the intention to die compared to those without the intention to die represented a significantly higher percentage in the 11–14-year-old age group (54.5%) compared to the older age group (27.5%, *p* < 0.034) and in girls (36%) compared to boys (15.3%, *p* < 0.04).

### 3.6. Risk Factors for Suicide Attempts

Suicide attempts in the past and self-harm behavior were not associated with a new suicide attempt. Stressful events related to suicide attempts were bullying (43.5%, *p* < 0.001), internet addiction (62.9%, *p* < 0.001), and quarreling with friends (35.5%, *p* < 0.001). The family’s socioeconomic status did not correlate with the children’s suicide attempts.

**Figure 4:**
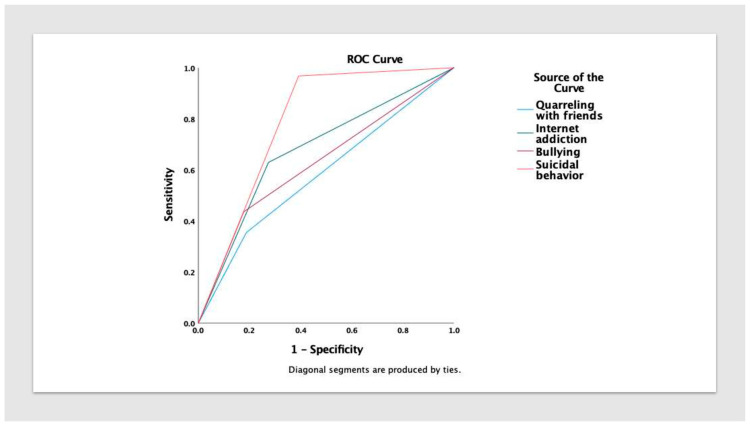
Area under the receiver operating characteristic curve (AUROC) for predicting suicide attempts in children and adolescents. Independently, suicidal behavior (0.79 (95% CI = 0.73–0.84), *p* < 0.001), internet addiction (0.68 (95% CI = 0.60–0.76), *p* < 0.001), and bullying (0.63 (95% CI = 0.55–0.71), *p* < 0.002) achieved the best AUROC.

In an ROC analysis, suicidal behavior (sensitivity 0.97, specificity 0.61), internet addiction (sensitivity 0.63, specificity 0.73), and bullying (sensitivity 0.44, specificity 0.82) showed the strongest independent predictive ability for a suicide attempt (Figure 4).

Suicidal behavior (0.79 (95% CI = 0.73–0.84), *p* < 0.001), internet addiction (0.68 (95% CI = 0.60–0.76), *p* < 0.001), and bullying (0.63 (95% CI = 0.55–0.71), *p* < 0.002) achieved the best AUROC. Quarreling with friends was a weaker predictor of a suicide attempt (0.58 (95% CI = 0.49–0.67), *p* < 0.049).

## 4. Discussion

Our study demonstrated a longitudinal increase in annual rates of suicidality, including active suicidal ideation and suicide attempts, among patients admitted to an academic PPC. Female adolescent patients suffered mainly from suicidal ideation and were predominantly admitted for attempted suicide or active suicide ideation, whereas males were for behavioral disorders and psychosis. In our cohort of patients, we showed that suicide attempts in the past and self-harm behavior are strongly associated with suicidality, whereas the risk factors independently associated with suicidality are friend quarreling, bullying, and internet addiction. We also showed that suicidal behavior, internet addiction, and bullying are independent predictors of a suicide attempt among children and adolescents admitted to a Pediatric Intensive Care Unit or a Pediatric Emergency Department and hospitalized in an academic PPC. Unexpectedly, younger patients presented self-harm behavior with the intention to die more often compared to older adolescents.

Pediatric and adolescent patients with mental and behavioral disorders, commonly suicidality and aggressiveness, have doubled over the past 15 years. Stressors occurring over the lifespan and unhealthy family functioning play a key role in bringing psychopathology up to the surface, depicted in our study. Family functioning expresses the ability of a family to meet the physical and emotional needs of the family’s members, being the most direct and predominant environment of a child’s development [23]. Children and adolescents with healthy family functioning and strong family cohesion are at a low risk of developing anxiety and withdrawal behaviors. Negative family relationships, such as parent–child conflicts and divorces, increase the risk of anxiety and depressive symptoms among adolescents [24] and are associated with adolescents’ internalizing problems, emotional and behavioral problems, peer problems, and conduct problems [25]. Related to the severity of family dysfunction are comorbid depression and subthreshold depressive symptoms in anxious children and adolescents [26].

The high percentages of parental psychiatric disorders in this study are supported by recent evidence, indicating that active parental psychiatric symptomatology is an established genetic–environmental factor influencing offspring psychopathology [27]. The children of parents with a mental illness may present cognitive and behavioral difficulties, resulting in poor quality of life and unhealthy social relationships [28]. Importantly, these children are at-risk of developing the same illness as their parental mental illness [29], whereas half of the children of parents with a severe mental illness are at a higher risk of developing a severe psychiatric disorder [30].

Suicide ideation prevalence, although rare in childhood, increases in adolescence and is rapidly escalated after the progression of suicide ideation to suicidal behavior. A large proportion of patients in our series expressed active suicidal ideation (46.2) or attempted suicide (24.9). Additionally, more than half of females were admitted for attempted suicide (26.5%) or active suicide ideation (32%), whereas the main reasons for readmission to PPC were attempted suicide (27%) or suicidal behavior (22.4%). Similarly, among Canadian adolescents admitted to the PICU with serious self-harm injuries, females demonstrated a higher rate of suicide attempts and prior mental health care engagement in contrast to males, who were more likely to die by suicide [31]. In similar studies, the proportion of admitted adolescents with suicide attempts was 24.5% [32], whereas admissions with suicidal behavior varied between 39% and 47%, reaching 58% in Norway [33] and 78.2% in Australia [7]. The median prevalence of any lifetime self-reported suicide attempt was 10.5% across the 17 countries that participated in the European School Survey Project on Alcohol and Other Drugs (ESPAD) 2007 school survey [19]. The finding that children < 14-years-old jump from height or cut their wrists more often than adolescents has not been reported before. Unexpectedly, in this study, children <14-years-old exhibited self-harm behavior with the intention to die more often compared to older adolescents’ ways of attempting suicide. In contrast, 17.3% of admissions through the Public Prosecutor’s Office in this study compare favorably to the 33% of forced admissions in the Norwegian study [33].

Suicide in adolescence has been identified as a serious public health problem worldwide. In 27 EU countries, suicide is the second cause of death among young people aged 15–19. Worryingly, the suicide rate among adolescents has increased in recent years in the USA by 24%, especially among females aged 10–14-years-old [34]. In Sweden, child suicides increased by 2.2% in each successive year from 2000 to 2018 (mean age of 16 years) [35]. Increasing trends of suicide attempts, suicidal ideation, and suicidality rates were also recorded during this 4-year study, more frequently among females and patients with self-harm behavior. These results are similar to those of a nationwide population-based study in Korea, revealing a 35.6% increase in the annual percentage change in the incidence rate of suicide-attempt-related emergency department visits over a 4-year period [36]. They have also reported that the incidence rate increase was higher among females and increased faster in mid-adolescence patients. Worryingly, during the three years of the COVID-19 pandemic (2019–2021), the number of suicide attempts by children and adolescents up to 18 years of age increased [37] along with an upward longitudinal trend of acute psychopathological symptoms, including depression, post-traumatic-stress disorder, and psychosis [10].

Stressful events related to internet addiction, bullying, and quarreling with friends were recognized as the most common predicting factors implicated in suicide attempts in this study. Similarly, in New Zealand, interpersonal conflict and relationship difficulties accounted for 50% of serious suicide attempts among young people aged 13–24, and only 6% related to school problems [38]. In our study, bullying, internet addiction, friends quarreling, and family problems were independent risk factors associated with suicidality. In a recent study among middle school students, independent risk factors associated with lifetime suicidal ideation and attempts included substance abuse and bullying victimization at school or electronically [39]. Importantly, cyberbullying experiences have recently been shown to be associated with suicidality in early adolescence [40].

Our findings that during early adolescence, suicidal children engage in problematic internet use confirm the results of a recent study showing that the problematic use of the internet and social media is independently associated with suicide attempts in young people [41]. Analyzing the changes in suicide rates after the release of “13 Reasons Why”, it is now assumed that suicide increases in youth only, especially in young females, which is consistent with a contagion by media [42]. Importantly, in this and previous studies, socioeconomic status did not relate to suicide attempts, although recent studies have shown a significant impact of the financial status, fiscal austerity, and hopelessness on suicidal behavior [43,44].

The main limitations of this study are its retrospective design and the short longitudinal duration. Although the sample size is not sufficiently large to permit definitive and generalized conclusions, it is homogenous, clearly depicting various dimensions of the problem in a high-risk cohort. Future research on stressful life events and associated suicidality in children in expanded geographic areas and in the post-COVID-19 period is needed.

## 5. Conclusions

In the current study, we estimated the prevalence of suicidality in pediatric patients admitted to an academic Pediatric Psychiatric Clinic and analyzed social and environmental risk factors associated with suicidality. We demonstrated a longitudinal increase in active suicidal ideation and annual suicide attempt rates among patients admitted to the clinic. Female adolescent patients suffered mainly from active suicidal ideation and were predominantly admitted to the Pediatric Psychiatric Clinic, with a tenth of them to the Pediatric Intensive Care Unit, for attempted suicide or active suicidal ideation, whereas males for behavioral disorders and psychosis. We found that suicide attempts in the past and self-harm behavior were strongly associated with suicidality, whereas the risk factors independently associated with suicidality were friend quarreling, bullying, and internet addiction. We also showed that suicidal behavior, internet addiction, and bullying were independent predictors of a suicide attempt among children and adolescents. Unexpectedly, we found that younger patients presented self-harm behavior with the intention to die more often compared to older adolescents. These findings have implications for prevention and intervention programs for children and adolescents, pointing to the need for further evaluation of specific aspects of family and social life, including the COVID-19 pandemic lockdown response.

## Figures and Tables

**Table 1:** Characteristics of acute mental health crisis episodes needing hospitalization in Pediatric Psychiatric Clinic.

Admission Characteristics		Age Group (Years)			* p
	Total	5–10	11–14	15–18	
*Admission*	*n (%)*				
First Admission	152 (61)	3 (2)	50 (32.9)	99 (65.1)	0.242
Readmission	97 (39)	0 (0.0)	27 (27.8)	70 (72.2)	
*Origin*					
From the Emergency Department	162 (65.1)	2 (1.2)	49 (30.3)	111 (68.5)	0.403
Another Clinic	49 (19.7)	1 (2)	17 (34.7)	31 (63.3)	
From Other Hospital	27 (10.8)	0 (0.0)	5 (18.5)	22 (81.5)	
From Outpatient Institutions	11 (4.4)	0 (0.0)	6 (54.5)	5 (45.5)	
*Intra-Hospital Transport*					
Pediatric Intensive Care Unit	5 (10.2)	0 (0.0)	3 (60)	2 (40)	0.003
Department of Pediatrics	23 (46.9)	1 (4.3)	14 (60.9)	8 (34.8)	
Department of Internal Medicine	12 (24.5)	0 (0.0)	0 (0.0)	12 (100)	
Department of Acute Psychiatry	9 (18.4)	0 (0.0)	0 (0.0)	9 (100)	
*Admission Modality*					
Voluntary	205 (82.6)	3 (1.5)	67 (32.7)	135 (65.9)	0.322
Public Prosecution Order	44 (17.3)	0 (0.0)	11 (23.3)	33 (76.7)	
*Reason for Hospitalization*					
Suicide Attempt	62 (24.9)	0 (0.0)	22 (35.5)	40 (64.5)	0.002
Suicidal Behavior ^#^	72 (28.9)	0 (0)	21 (29.2)	51 (70.8)	
Emotional Disorder	33 (13.3)	1 (3.0)	8 (24.2)	24 (72.7)	
Behavioral Disorder	33 (13.3)	2 (6.1)	9 (27.3)	22 (66.7)	
Eating Disorder	8 (3.2)	0 (0.0)	8 (100)	0 (0.0)	
Social Reason	11 (4.4)	0 (0.0)	4 (36.4)	7 (63.6)	
Psychosis	30 (12.0)	0 (0.0)	5 (16.7)	25 (83.3)	

* Among age groups (ANOVA test), ^#^ evidenced at home, school, or other social environments or media.

**Table 2:** Patients’ demographics, school, family, and environmental problems, acute stressful events, and particular influencing problems.

		Age Group (Years)			* p
	Total	5–10	11–14	15–18	
(First admission data, *n* = 152)	*n* (%)				
*Characteristics of patients*					
Total number of patients	152 (100)	3 (2)	50 (32.9)	99 (65.1)	
Females/males	107/45 (70.4/29.6)	1/2 (0.9/4.4)	37/13 (34.6/28.9)	69/30 (64.5/66.7)	0.331
School abandonment	30 (21.5)	1 (3.3)	7 (23.3)	22 (73.3)	0.156
*Family Environment*					
Divorced parents	37 (30.6)	1 (2.7)	15 (40.5)	21 (56.8)	0.938
Psychiatric diseases of father or suspicion	52 (35.1)	1 (1.9)	20 (38.5)	31 (59.6)	0.423
Psychiatric diseases of mother or suspicion	98 (65.0)	1 (1)	41 (41.8)	56 (57.1)	0.018
Father’s addictions	23 (15.5)	0 (0.0)	10 (43.5)	13 (56.5)	0.423
Institutions (orphanages)	22 (14.6)	0 (0.0)	6 (27.3)	16 (72.7)	0.314
(All admissions data, *n* = 249)	*n* (%)				
*Recent Stressful Events*					
Socio-economic status low	109 (72.7)	2 (1.8)	41 (37.6)	66 (60.6)	0.193
Problems with family relationships	77 (30.9)	1 (1.3)	24 (31.2)	52 (67.5)	0.994
Problems with school	22 (13.2)	0 (0.0)	8 (36.4)	14 (63.6)	0.819
Quarreling with friends	57 (22.9)	0 (0.0)	19 (33.3)	38 (66.7)	0.593
Bullying	60 (24.1)	0 (0.0)	26 (43.3)	34 (56.4)	0.042
Child abuse: physical	25 (10.0)	1 (4.0)	7 (28.0)	17 (68)	0.557
Child abuse: sexual	15 (6.0)	0 (0.0)	5 (33.3)	10 (66.7)	
*Patient’s Conditions/Addictions*					
Chronic disease	68 (27.8)	1 (1.5)	20 (29.4)	47 (69.1)	0.927
Psychotic episode	50 (20.2)	0 (0.0)	12 (24)	38 (76)	0.303
Aggressive behavior	27 (10.9)	0 (0.0)	4 (14.8)	23 (85.2)	0.660
Internet addicted (patient)	91 (36.5)	2 (2.2)	28 (30.8)	61 (67.0)	0.552
Substance abuse: alcohol	6 (2.5)	0 (0.0)	1 (16.7)	5 (83.3)	0.338
Substance abuse (patient): cannabinoids	32 (13.1)	0 (0.0)	5 (15.6)	27 (84.4)	
Substance abuse (patient): other and/or more substances	6 (2.5)	0 (0.0)	1 (16.7)	5 (83.3)	

* Among age groups (ANOVA test).

**Table 3:** Suicidality, suicidal ideation, suicide attempts, and methods (all admissions).

		Age Group (Years)			* *p*
	Total	5–10	11–14	15–18	
	*n (%)*				
*Suicidality*					
Suicide attempt	62 (24.9)	0 (0.0)	22 (35.5)	40 (64.5)	0.430
Active suicidal ideation	115 (46.2)	2 (1.7)	34 (29.6)	79 (68.7)	0.721
Suicidality (both)	177 (71.1)	2 (1.1)	56 (31.6)	119 (67.2)	0.920
*Suicide attempt*					
First attempt at admission	43 (28.3)	0 (0.0)	16 (37.2)	27 (62.8)	0.450
Violent	23 (37.1)	0 (0.0)	12 (52.2)	11 (47.8)	0.034
Not violent	39 (62.9)	0 (0.0)	10 (25.6	29 (74.4)	
Attempt at readmission	19 (19.6)	0 (0.0)	6 (31.6)	13 (68.4)	0.560
Previous attempts ^	47 (31.3)	0 (0.0)	14 (29.8)	33 (70.2)	0.377
*Suicide attempt method*					
Drug overdose	35 (56.5)	0 (0.0)	9 (25.7)	26 (74.3)	0.134
Jumping from height	8 (12.9)	0 (0.0)	5 (62.5)	3 (37.5)	
Poison	4 (6.5)	0 (0.0)	1 (25)	3 (75)	
Firearm	2 (3.2)	0 (0.0)	1 (50)	1 (50)	
Wrist-cutting	3 (4.8)	0 (0.0)	3 (100)	0 (0.0)	
Drowning	2 (3.2)	0 (0.0)	0 (0.0)	2 (100)	
Vehicular impact	4 (6.5)	0 (0.0)	1 (25.0)	3 (75)	
Hanging	2 (3.2)	0 (0.0)	1 (50)	1 (50)	
Self-strangulation	1 (1.6)	0 (0.0)	1 (100)	0 (0.0)	
Self-immolation	1 (1.6)	0 (0.0)	0 (0.0)	1 (100)	

* Among age groups (ANOVA test), ^ before first admission.

## Data Availability

Clinical data presented in the study are all contained within this article. The institution’s rights, including legal and ethical concerns, patient privacy, and confidentiality, restrict access to detailed data sharing.
